# F193. DYSREGULATION OF RETINOID SIGNALLING IN SCHIZOPHRENIA OBSERVED IN WHOLE GENOME SEQUENCE ANALYSIS

**DOI:** 10.1093/schbul/sby017.724

**Published:** 2018-04-01

**Authors:** William Reay, Joshua Atkins, Chantel Fitzsimmons, Melissa Green, Vaughn Carr, Murray Cairns

**Affiliations:** 1 University of Newcastle; 2 School of Psychiatry, University of New South Wales

## Abstract

**Background:**

Retinoic acid (RA) is intrinsically linked to neurodevelopment and has been implicated in schizophrenia (SZ). This is supported by preliminary trials of a retinoid receptor agonist, Bexarotene, as an adjuvant and the association of five common variants in proximity to members of this pathway at genome wide significance. In addition to these high frequency variants with small effect size, we suspect that this pathway is also affected by rare variants with much higher impact. We aimed to examine the burden of variants in retinoid loci in schizophrenia along with its potential consequences for clinical practice.

**Methods:**

Whole genome sequencing was performed on SZ cases (N=331) and non-psychiatric controls (N=167). Cases were further clustered by cognitive measures to derive the cognitive deficit (CD; N=166) and cognitively spared (CS; N=165) subtypes. Disease and subtype associated genomic variation was then analysed in a panel of 129 genes selected for involvement in RA biology (Molecular Signatures Database). Rare variation was aggregated at the gene level using the optimal unified sequence kernel association test (SKAT-O). Clinical metadata was further examined for each case with a rare putative high impact loss of function variant in a RA panel gene predicted using SnpEff. The rare variant burden on target genes of RA receptor binding in SZ was investigated by logistic regression of variants mapped to consensus 5 base pair spaced direct repeat (DR5) retinoic acid response element (RARE) motifs.

**Results:**

Gene level rare variant association uncovered suggestive associations with SZ (P < 0.01) for three retinoid genes – RBP3, ADH1C and RPE65. In addition, a stronger signal was detected overall for CD cases implicating four additional genes including the RA receptors RARB (P = 1.1 x 10–3) and RARG (P = 9.2 x 10–3). SZ patients with a rare high impact genotype predicted in a RA panel gene were more likely to have serious symptomology as defined by a global assessment of functioning (GAF) score below 50, P = 7.1 x 10–3 (Two-Tailed Fisher’s Exact Test). We also found evidence of an increased burden of rare variants within predicted DR5-RARE in SZ (P = 0.017, odds ratio [OR] = 1.094, 95% confidence interval [CI] = 1.023- 1.186), however, there was no significant difference between the cognitive subtypes (CD/CS, P = 0.8, OR = 1.002, 95% CI = 0.961–1.045).

**Discussion:**

Our findings suggest that RA mediated control of gene expression is heterogeneously disrupted in SZ by rare variants in DR5-RARE motifs. Strong signals in the context of our sample size further support the possibility of enrichment of rare loci in genes involved with RA biology, particularly in CD cases with impaired cognition. Moreover, we identified a subset of patients with likely high effect size genotypes in the RA pathway. Future work will examine whether these high-risk patients would benefit from retinoid based pharmacological intervention.

